# Association between preoperative phase angle and all‐cause mortality after cardiovascular surgery: A retrospective cohort study

**DOI:** 10.1002/jcsm.13514

**Published:** 2024-06-10

**Authors:** Kenichi Shibata, Masataka Kameshima, Takuji Adachi, Hisako Kito, Chikako Tanaka, Taisei Sano, Mizuki Tanaka, Yoriyasu Suzuki, Mototsugu Tamaki, Hideki Kitamura

**Affiliations:** ^1^ Department of Cardiac Rehabilitation Nagoya Heart Center Nagoya Japan; ^2^ Department of Integrated Health Sciences Nagoya University Graduate School of Medicine Nagoya Japan; ^3^ Department of Cardiology Nagoya Heart Canter Nagoya Japan; ^4^ Department of Cardiovascular Surgery Nagoya Heart Canter Nagoya Japan

**Keywords:** all‐cause mortality, cardiovascular surgery, phase angle, physical function

## Abstract

**Background:**

The importance of preoperative physical function assessment for post‐operative intervention has been reported in older patients undergoing cardiovascular surgery. Phase angle (PhA), measured using bioelectrical impedance analysis, is an indicator of cellular health and integrity and is reported as a prognostic factor in several chronic diseases; however, its association with the long‐term prognosis of cardiovascular surgery remains unclear. This study aimed to investigate the prognostic value of PhA for long‐term mortality in patients undergoing cardiovascular surgery.

**Methods:**

This retrospective cohort study included consecutive patients who underwent elective cardiovascular surgery between October 2016 and March 2021 at Nagoya Heart Center, Japan. PhA was assessed using bioelectrical impedance analysis before surgery, and physical function measures (gait speed, grip strength and short physical performance battery [SPPB]) were measured synchronously. The association between PhA and all‐cause mortality after discharge was assessed using Kaplan–Meier and multivariate Cox regression analyses. The incremental prognostic value of PhA was compared with other physical function measures using net reclassification improvement (NRI) and integrated discrimination improvement (IDI).

**Results:**

A total of 858 patients were included in the present analysis (mean age = 68.4 ± 11.9 years, 67.6% male). PhA positively correlated with body mass index (*ρ* = 0.38, *P* < 0.001), skeletal muscle mass index (*ρ* = 0.58, *P* < 0.001), usual gait speed (*ρ* = 0.44, *P* < 0.001), grip strength (*ρ* = 0.73, *P* < 0.001) and SPPB (*ρ* = 0.51, *P* < 0.001). The mean follow‐up period, within which 44 (4.7%) died, was 908.9 ± 499.9 days for the entire cohort. Kaplan–Meier survival curves based on the PhA tertiles showed that higher PhA was associated with better survival (log‐rank test, *P* < 0.001). The Cox regression analysis showed the independent association of PhA with mortality risk (hazard ratio: 0.91 per 0.1° increment; 95% confidence interval [CI]: 0.87–0.95; *P* < 0.001). The NRI and IDI showed significant improvements in predicting mortality after adding PhA to the clinical model consisting of age, sex and cardiac and renal function (NRI: 0.426, 95% CI: 0.124–0.729, *P* = 0.006; IDI: 0.037, 95% CI: 0.012–0.062, *P* = 0.003). The predictive model consisting of the clinical model and PhA was superior to the model consisting of the clinical model and each of the other physical function indicators (*P* < 0.05).

**Conclusions:**

PhA correlated with physical function and independently predicted long‐term mortality after cardiovascular surgery. The additive prognostic value of PhA compared with the other physical function measures suggests the clinical usefulness of preoperative PhA for risk stratification in planning post‐operative treatment and rehabilitation.

## Introduction

Owing to an aging population, the number of older patients undergoing cardiovascular surgery is increasing globally. Physical frailty, a prevalent clinical syndrome in old age, is a known risk factor for unfavourable clinical outcomes,^1^ and perioperative and post‐operative management and rehabilitation have become major clinical concerns in older patients. The guidelines for cardiac rehabilitation have recommended that geriatric assessment include physical and cognitive function in routine practice. A recent study showed that routine preoperative frailty assessment of patients undergoing cardiovascular surgery resulted in an 18% reduction in 1‐year mortality,^2^ suggesting the clinical significance of preoperative physical assessment in planning interventions.

To date, physical function assessment has focused on performance, such as functional mobility and muscle strength.^3^ Added to this, there has been a growing interest in the association between body composition and perioperative clinical outcomes in the past decade.^4^ Bioelectrical impedance analysis (BIA) is a safe, non‐invasive and repeatable method to assess body composition. A previous study reported that BIA‐estimated skeletal muscle mass was associated with muscle strength and post‐operative outcomes, especially when coexisting with high fat mass.^5^ Adipose tissue volume calculated by BIA has also been reported to predict obesity and metabolic syndrome in patients undergoing cardiovascular surgery or percutaneous coronary intervention.^6^ Phase angle (PhA) is another BIA parameter calculated as the angle representing the ratio of reactance of the cell membrane to resistance of the body water, a raw parameter derived from bioimpedance analysis. PhA is thought to reflect cellular integrity, and a higher value indicates better physical health, including muscle quality.^7^ Low PhA is associated with many age‐related health conditions, such as malnutrition, sarcopenia, frailty and an increased risk of disability incidence.^8^

Moreover, lower PhA values have been reported to be associated with a worse prognosis in several chronic diseases, including heart failure.^9^ In addition to such chronic conditions, PhA can also be measured in hospitalized patients with symptomatic activity limitations, making it a useful clinical indicator.^10^ However, the evidence regarding the association between the PhA and long‐term prognosis after cardiovascular surgery is limited. A previous study reported that a low PhA was a predictor of post‐operative mortality^11^; however, confounding factors such as age, sex and cardiac or renal function were not fully considered, and only short‐term mortality at 12 months of follow‐up was examined. Furthermore, no reports have compared the prognostic values of PhA and general physical function measurements. If the prognostic information of PhA is comparable to or greater than that of other physical function indicators, it can serve as a safe and feasible preoperative physical assessment for risk stratification and the planning of tailored interventions. Therefore, this study aimed to investigate the effects of PhA on the long‐term mortality of patients undergoing cardiovascular surgery.

## Methods

This retrospective cohort study included 941 consecutive patients who underwent elective cardiovascular surgery, including coronary artery bypass surgery, valve replacement or repair, coronary artery bypass surgery with concomitant valve replacement or repair and aortic surgery, between October 2016 and March 2021 at Nagoya Heart Center, Japan. Trained cardiac surgeons were responsible for the diagnosis and the decision on the procedure, with instructions for preoperative evaluation and post‐operative rehabilitation. All patients received inpatient cardiac rehabilitation consisting of an early post‐operative mobilization programme, exercise training and disease management instructions for discharge from the day after surgery until discharge. In addition, patients who were able to participate underwent an outpatient cardiac rehabilitation programme at the hospital after discharge. The outpatient cardiac rehabilitation programme was initiated approximately 1 week post‐discharge and continued for 3–6 months. The programme followed guidelines and included supervised exercise sessions at the hospital (1–3 times per week, 60 min per session; ergometer cycling, treadmill walking and resistance training), educational classes, personal counselling and home exercise.^3^ The exclusion criteria were as follows: (1) patients with cardiovascular implantable electronic devices (contraindication to bioimpedance testing); (2) patients with thoracic aortic disease treated with endovascular aneurysm repair and with abdominal aortic aneurysm; and (3) patients who died in the hospital. Within 1 month prior to surgery, trained physical therapists performed BIA and physical function measurements. Variables such as age, sex, height and weight measurements, comorbidities and clinical data were also retrieved.

The study protocol was in compliance with the principles of the Declaration of Helsinki and was approved by the Research Ethics Committee of the Nagoya Heart Center (Approval No. NHC2022‐1004‐09; approval date: 4 October 2022). The requirement for written informed consent was waived by the Research Ethics Committee of the Nagoya Heart Center because the study met the conditions outlined in the Japanese Ethical Guidelines for Medical and Biological Research Involving Human Subjects. Instead, all patients were informed about their participation in the study, and each patient was offered an opportunity to opt out. Information regarding this study, such as the inclusion criteria and the opportunity to opt out, was provided on the hospital's website. No patients had opted out of the study at the time of analysis.

### Bioelectrical impedance analysis

To measure preoperative PhA, BIA was performed using an InBody 770 BIA unit (InBody Co., Ltd., Seoul, Korea). The measurements were conducted as described in a previous study.^12^ After 3 min of rest, the patient assumed a standing position on a platform with both hands on the handles of the BIA device and the soles of the hands and feet in contact with a series of electrodes.^12^ This procedure allows multifrequency measurements and can measure not only whole‐body PhA but also segmental PhA.

In this study, we investigated the whole‐body PhA at 50 kHz, which is the most commonly used frequency.^12^, ^13^ The whole‐body and segmental PhAs were calculated with reactance (Xc) and resistance (R) by the BIA device according to the following formula: PhA (°) = arctangent (Xc/R) × (180°/π). To avoid confounding the timing of measurements, BIA measurements were taken in the afternoon of the day before or 2 days before surgery. Skeletal muscle mass index (SMI) was calculated by dividing the estimated appendicular skeletal muscle mass (kilograms) by the square of the height (square metres).

### Measurements of physical function

Preoperative physical function was assessed at the time of the BIA. Physical function measurements included gait speed, grip strength and short physical performance battery (SPPB). Gait speed is a simple and highly reliable indicator of physical function and mobility.^14^ Gait speed is also known as a predictor of life expectancy in older individuals^15^ and an indicator of post‐operative prognosis.^16^ A 10‐m walkway was used to measure the usual gait speed, and the faster value of the two measurements was used for the analysis. Grip strength is widely used for screening muscle strength and has been reported to be associated with prognosis after cardiac surgery.^17^ Grip strength was assessed with a Jamar dynamometer (Jamar Plus+; Sammons Preston, Bolingbrook, IL, USA) and measured according to reliable methods reported in a previous study.^18^ The Jamar dynamometer was used in the second handle position, and the patient was measured in a sitting position with the wrists in neutral and the elbows flexed at 90°. Two trials were completed for each hand, and the highest value was used for the analysis. Considering sex differences in muscle strength, low grip strength was defined as grip strength of <28 kg in men and <18 kg in women based on the Asian Working Group for Sarcopenia criteria of 2019.^19^ The SPPB comprises balance, gait and five‐time sit‐to‐stand tests.^20^ The balance test evaluates the ability of the patient to stand with both feet side by side in the semi‐tandem and tandem positions. The gait test assesses the time to walk 4 m at the patient's usual pace. Patients could use assistive devices if needed. The five‐time sit‐to‐stand test measured the time to rise from a chair five consecutive times as quickly as possible with the arms folded across the chest. The total SPPB scores range from 0 to 12, with higher scores indicating better physical function. The SPPB score has also been reported to be associated with prognosis in a systematic review.^21^

### Clinical data

Information on patient age, sex, body mass index (BMI), medical history and clinical data, namely, results of echocardiography and blood biochemistry tests, was retrieved from the medical records. The estimated glomerular filtration rate (eGFR) is calculated by the following GFR equation adjusted for the Japanese population: eGFR (mL/min/1.73 m^2^) = 194 × Cr^−1.094^ × Age^−0.287^ (×0.739 if female).^22^ The geriatric nutritional risk index (GNRI) was calculated based on a previous study.^23^ All preoperative clinical data were obtained or measured between the day before surgery and the day of surgery. The type and duration of the surgery were obtained from the surgical records.

### Statistical analysis

Patients with missing preoperative BIA data were excluded from the analysis. Continuous variables with or without a normal distribution were expressed as means ± standard deviations (SDs) or medians with interquartile ranges. Correlations between the PhA and physical function were assessed using Spearman's correlation analysis. As some patients had missing data for physical function measurements, a pairwise deletion was performed.

To explore the factors associated with preoperative PhA, we performed multivariate linear regression analysis using PhA as the dependent variable. Independent variables selected that have been reported to be associated with PhA in the general older population were cardiac function, renal function and nutritional status^8^, ^24^, ^25^; physical function was not included due to multicollinearity. The multivariate model included patient characteristics related to cardiovascular disease and comorbidities, including cancer and diabetes.

The Kaplan–Meier method was used to generate event‐free survival curves based on PhA tertiles, and differences in mortality were assessed using the log‐rank test. In addition, the cut‐off value for all‐cause mortality at PhA was calculated using the receiver operating characteristic curve. Univariate Cox regression analysis was performed to obtain the hazard ratio (HR) for all‐cause mortality during the follow‐up period. Multivariate analysis was then performed using the baseline clinical characteristics and other variables with a univariate *P*‐value of <0.05 to examine the independent association of PhA with all‐cause mortality. Considering the number of adjustment variables, two prediction models were constructed: Cox Model 1 included age, sex, left ventricular ejection fraction (LVEF) and eGFR as adjustment variables, and Cox Model 2 included age, sex, LVEF, eGFR, log N‐terminal pro‐B‐type natriuretic peptide (NT‐proBNP) and GNRI.

The number of variables to be included was determined based on the concept of 10 outcomes for one independent variable in Cox regression^26^ and 15 subjects for one independent variable in linear regression.^27^

Improvements in the predictive accuracy of PhA compared with the conventional prediction of survival using the clinical model and physical function assessment were determined by calculating the net reclassification improvement (NRI) and integrated discrimination improvement (IDI) based on a logit model.^28^ The clinical model included age, sex and cardiac and renal function.

All statistical analyses were performed using EZR (Saitama Medical Center, Jichi Medical University, Saitama, Japan), which is a graphical user interface for R (R Foundation for Statistical Computing, Vienna, Austria).^29^ All tests with two‐sided *P*‐values < 0.05 were considered significant.

## Results

**Figure 1:**
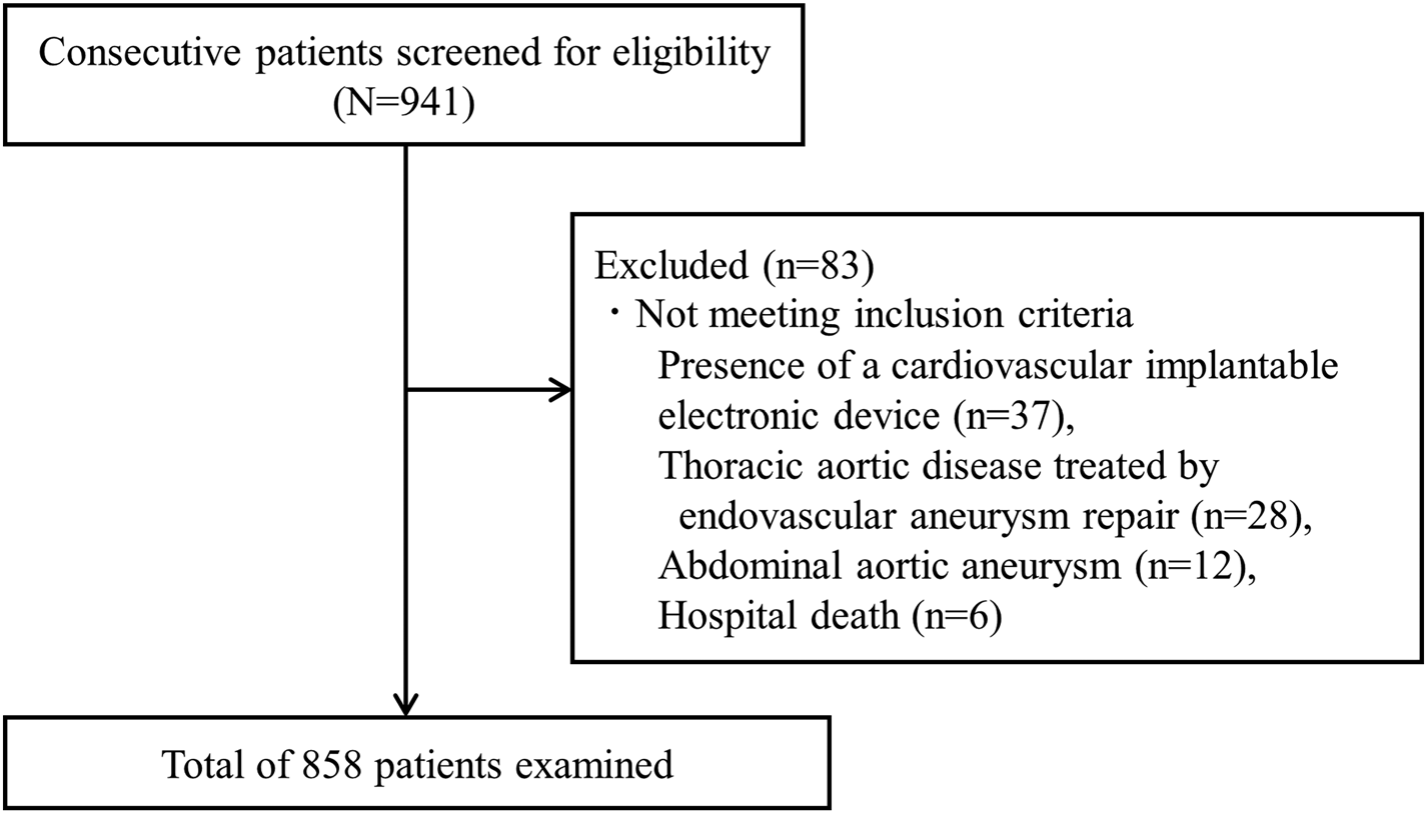
Study flow diagram.

**Figure 2:**
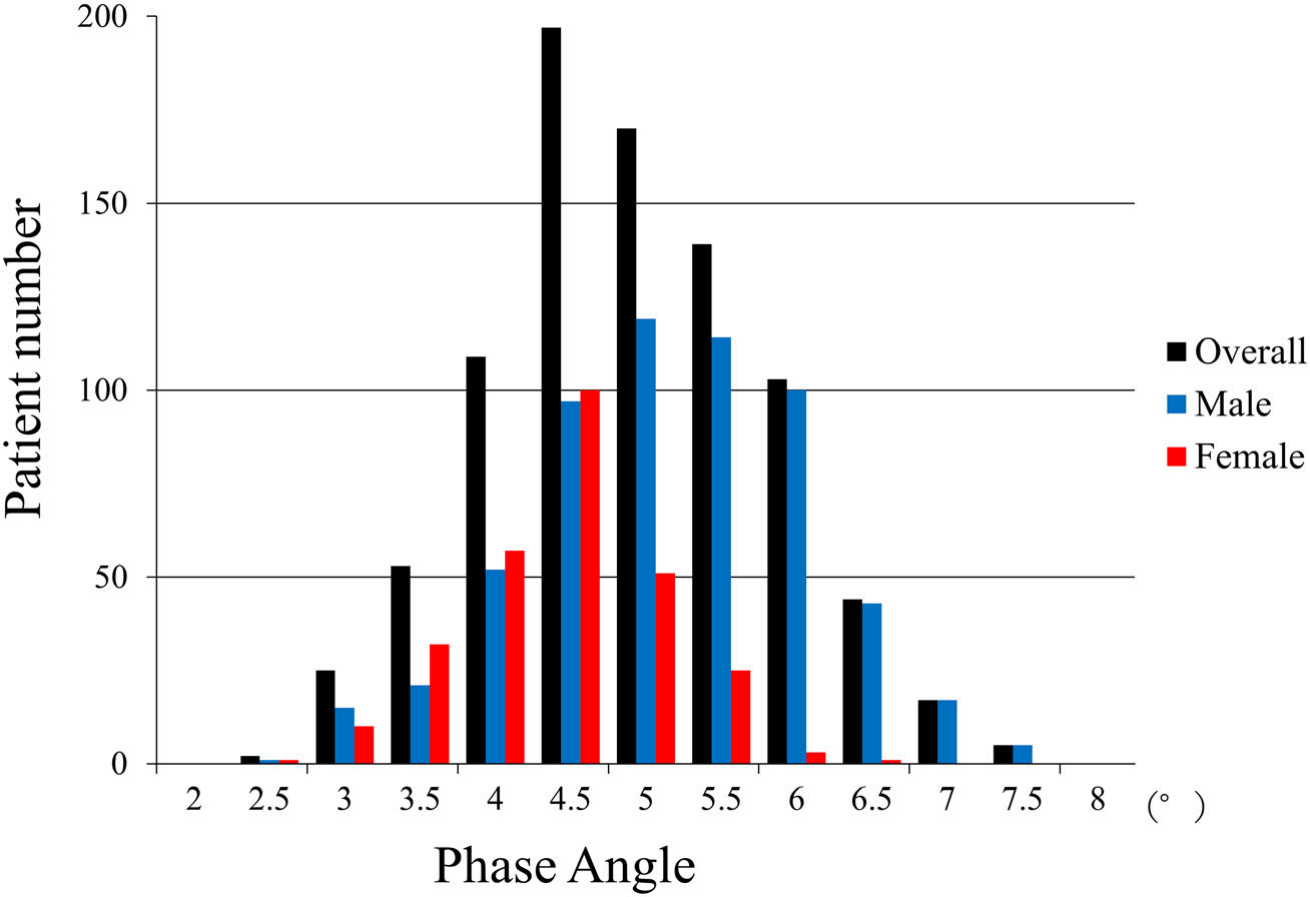
Distributions of phase angle in the overall, male and female populations.

Of the 941 patients who underwent elective cardiovascular surgery during the study period, 83 patients were excluded, including 37 patients with a cardiovascular implantable electronic device, 28 patients who were treated by endovascular aneurysm repair, 12 patients who had an abdominal aortic aneurysm and 6 patients who died in‐hospital. Finally, 858 were included in the analysis (*Figure* *1*). The baseline patient characteristics are shown in *Table* *1*. The average age was 68.4 ± 11.9 years, and 67.6% of the patients were male. The distribution of PhA in the overall male and female patient populations is shown in *Figure* *2*.

**Table 1:** Baseline characteristics of study patients

	Overall, *n* = 858
Baseline clinical characteristics	
Age, years	68.4 ± 11.9
Male, *n*	580 (67.6%)
Height, cm	161.5 ± 9.3
Weight, kg	60.1 ± 13.2
Body mass index, kg/m^2^	22.9 ± 3.9
SMI, kg/m^2^	6.8 ± 1.2
LVEF, %	54.9 ± 14.0
Physical function	
10‐m gait speed (m/s, *n* = 856)	1.09 ± 0.26
<1.0 m/s, *n*	289 (34.8%)
SPPB (point, *n* = 845)	12 (10–12)
≤9, *n*	168 (20.5%)
Peak grip strength (kgf, *n* = 851)	29.3 ± 10.4
Male < 28 kgf and female < 18 kgf, *n*	253 (29.7%)
Walking aids, *n*	
None	840 (97.9%)
T‐cane	10 (1.2%)
Other	8 (0.9%)
Preprocedural laboratory data	
eGFR, mL/min/1.73 m^2^	54.1 ± 22.7
Albumin, g/dL	4.0 ± 0.4
Haemoglobin, g/dL	13.4 ± 1.8
NT‐proBNP, pg/mL	528.0 (134.8–1950.0)
Comorbidities	
Diabetes mellitus, *n*	277 (32.3%)
Hypertension, *n*	559 (65.2%)
Cancer, *n*	98 (11.4%)
Prior heart failure, *n*	248 (28.9%)
Prior open‐heart surgery, *n*	30 (3.5%)
Type of operation, *n*	
CABG	236 (27.5%)
Valve	385 (44.9%)
Aortic	48 (5.6%)
Concomitant	167 (19.5%)
Other	22 (2.5%)
Nutritional status	
GNRI	98.6 ± 7.4
Duration of surgery, min	255.7 ± 84.5
Outpatient cardiac rehabilitation, *n*	201 (23.4%)
Medications, *n*	
Beta‐blocker	354 (41.3%)
ACE‐i or ARB	403 (47.0%)
Diuretics	315 (36.7%)
Statins	354 (41.3%)
Bioelectrical impedance analysis	
Phase angle, °	4.8 ± 0.9
In male (°, *n* = 580)	5.0 ± 0.9
In female (°, *n* = 278)	4.2 ± 0.7

*Note*: Values are numbers (%), mean ± SD or median with interquartile range. Abbreviations: ACE‐i, angiotensin‐converting enzyme inhibitor; ARB, angiotensin II receptor blocker; CABG, coronary artery bypass grafting; eGFR, estimated glomerular filtration rate; GNRI, geriatric nutritional risk index; LVEF, left ventricular ejection fraction; NT‐proBNP, N‐terminal pro‐B‐type natriuretic peptide; SMI, skeletal muscle mass index; SPPB, short physical performance battery.

The correlations between PhA and body composition, physical function indicators and age are presented in *Table* *2*. PhA was positively correlated with BMI (*ρ* = 0.38, *P* < 0.001), SMI (*ρ* = 0.58, *P* < 0.001), usual gait speed (*ρ* = 0.44, *P* < 0.001), SPPB (*ρ* = 0.51, *P* < 0.001) and grip strength (*ρ* = 0.73, *P* < 0.001) and negatively correlated with age (*ρ* = −0.57, *P* < 0.001).

**Table 2:** Correlations between phase angle and body composition, physical function and age

Variable	*ρ*	*P*‐value
BMI, kg/m^2^	0.38	<0.001
SMI, kg/m^2^	0.58	<0.001
10‐m gait speed, m/s	0.44	<0.001
SPPB	0.51	<0.001
Peak grip strength, kgf	0.73	<0.001
Age, years	−0.57	<0.001

*Note*: Missing data: 10‐m gait speed, *n* = 2; SPPB, *n* = 13; grip strength, *n* = 7. Abbreviations: BMI, body mass index; SMI, skeletal muscle mass index; SPPB, short physical performance battery.

*Table* *3* shows the results of the multiple linear regression analysis to examine the correlates of preoperative PhA. Age, LVEF, log NT‐proBNP levels and the presence of cancer and diabetes mellitus were negatively associated with PhA, whereas sex and the GNRI were positively associated with preoperative PhA (*Table* *3*).

**Table 3:** Linear regression analysis for phase angle

	Partial regression coefficient	Standardized regression coefficient (*β*)	95% CI	*P*‐value
Age (per 1‐year increase)	−0.027	−0.353	−0.400 to −0.305	<0.001
Male (for female)	0.561	0.291	0.246 to 0.335	<0.001
LVEF (per 1% increase)	−0.003	−0.054	−0.105 to −0.004	0.035
eGFR (per 1 mL/min/1.73 m^2^ increase)	−0.000	−0.002	−0.059 to 0.054	0.942
Log NT‐proBNP (per 1 increase)	−0.326	−0.306	−0.375 to −0.236	<0.001
GNRI (per 1 increase)	0.033	0.267	0.217 to 0.316	<0.001
Cancer	−0.148	−0.052	−0.095 to −0.010	0.016
Prior heart failure	0.039	0.020	−0.026 to 0.065	0.397
Diabetes mellitus	−0.136	−0.070	−0.114 to −0.027	0.001
Hypertension	−0.033	−0.017	−0.061 to 0.026	0.434

*Note*: Adjusted *R* ^2^ = 0.626. Abbreviations: CI, confidence interval; eGFR, estimated glomerular filtration rate; GNRI, geriatric nutritional risk index; LVEF, left ventricular ejection fraction; NT‐proBNP, N‐terminal pro‐B‐type natriuretic peptide.

**Figure 3:**
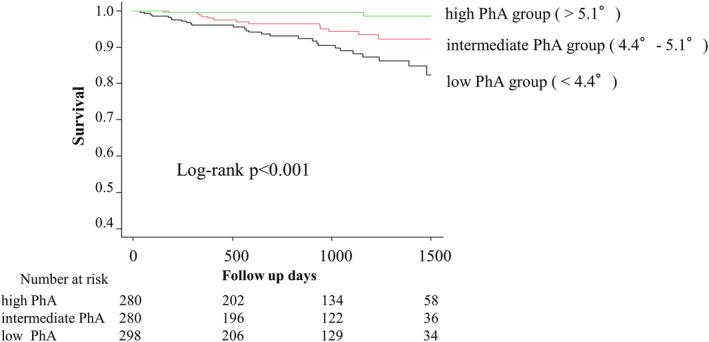
Kaplan–Meier survival curves for cumulative mortality after cardiovascular surgery in three groups divided by phase angle (PhA) tertiles.

The mean follow‐up period was 908.9 ± 499.9 days for the entire cohort, and 44 patients (4.7%; males: 31 patients, females: 13 patients) died during the follow‐up period. The Kaplan–Meier survival curves comparing the PhA tertiles are presented in *Figure* *3*. Patients in the high PhA group had a significantly better survival rate (log‐rank test, *P* < 0.001). The results of the cut‐off value from the receiver operating characteristic curve for all‐cause mortality were 4.5° (sensitivity, 0.795; specificity, 0.576; area under the curve, 0.758), and higher PhA had a significantly better survival rate (log‐rank test, *P* < 0.001) (*Figures* *S1* and *S2*).

The results of the Cox regression analysis are presented in *Table* *4*. In the univariate analysis, lower PhA, older age, lower LVEF, lower eGFR, lower GNRI and higher log NT‐proBNP levels were significantly associated with increased mortality after discharge. The association between PhA and all‐cause mortality was statistically significant, independent of age, sex, LVEF and eGFR (HR: 0.91 per 0.1° increment, 95% confidence interval [CI]: 0.87–0.95, *P* < 0.001). In addition, age‐adjusted analyses by sex showed that the HR for males was 0.87 per 0.1° increment (95% CI: 0.83–0.91, *P* < 0.001) and for females was 0.93 per 0.1° increment (95% CI: 0.85–1.02, *P* = 0.11).

**Table 4:** Cox regression analysis to examine the association between phase angle and all‐cause mortality

Explanatory variables	Univariate analysis			Multivariate analysis					
				Cox Model 1			Cox Model 2		
	HR	95% CI	*P*‐value	HR	95% CI	*P*‐value	HR	95% CI	*P*‐value
Phase angle (per 0.1° increase)	0.89	0.86–0.93	<0.001	0.91	0.87–0.95	<0.001	0.93	0.88–0.98	0.013
Adjusting factors									
Age (per 1‐year increase)	1.04	1.01–1.08	0.011	1.01	0.98–1.05	0.49	1.01	0.98–1.05	0.41
Male (for female)	1.20	0.63–2.29	0.59	1.98	0.98–3.97	0.056	1.87	0.89–3.93	0.099
LVEF (per 1% increase)	0.97	0.95–0.99	0.002	0.98	0.97–1.00	0.11	0.99	0.97–1.01	0.33
eGFR (per 1 mL/min/1.73 m^2^ increase)	0.97	0.95–0.98	<0.001	0.98	0.97–1.00	0.01	0.99	0.97–1.01	0.36
Log NT‐proBNP (per 1 increase)	3.41	2.31–5.04	<0.001	—	—	—	1.51	0.74–3.08	0.26
GNRI (per 1 increase)	0.92	0.89–0.95	<0.001	—	—	—	0.98	0.94–1.03	0.48
Cancer	0.58	0.18–1.88	0.37	—	—	—	—	—	—
CABG	0.98	0.51–1.91	0.96	—	—	—	—	—	—
Prior heart failure	0.97	0.51–1.85	0.92	—	—	—	—	—	—
Prior open‐heart surgery	0.78	0.11–5.63	0.80	—	—	—	—	—	—
Diabetes mellitus	1.51	0.83–2.75	0.18	—	—	—	—	—	—
Hypertension	0.95	0.52–1.76	0.88	—	—	—	—	—	—
Body mass index (per 1 kg/m^2^ increase)	0.93	0.85–1.01	0.08	—	—	—	—	—	—

Abbreviations: CABG, coronary artery bypass grafting; CI, confidence interval; eGFR, estimated glomerular filtration rate; GNRI, geriatric nutritional risk index; HR, hazard ratio; LVEF, left ventricular ejection fraction; NT‐proBNP, N‐terminal pro‐B‐type natriuretic peptide.

*Table* *5* shows the incremental values of the PhA and other physical function indicators for the predictive model, including age, sex and cardiac and renal function. The NRI and IDI showed significant improvements for predicting mortality when combining PhA and a clinical model consisting of age, sex and cardiac and renal function (NRI: 0.426, 95% CI: 0.124–0.729, *P* = 0.006; IDI: 0.037, 95% CI: 0.012–0.062, *P* = 0.003). Moreover, the predictive model consisting of the clinical model and PhA was superior to the model consisting of the clinical model and each of the other physical function indicators (*P* < 0.05).

**Table 5:** Net reclassification improvement and integrated discrimination improvement for comparison among physical functions

	NRI	95% CI	*P*‐value	IDI	95% CI	*P*‐value
Clinical model	Reference			Reference		
Clinical model + PhA	0.426	0.124 to 0.729	0.006	0.037	0.012 to 0.062	0.003
Clinical model + slow gait speed^a^	0.398	0.092 to 0.705	0.011	0.01	0.0037 to 0.017	0.002
Clinical model + low SPPB^b^	0.262	−0.055 to 0.579	0.106	0.011	0.001 to 0.02	0.03
Clinical model + SPPB	0.054	−0.263 to 0.371	0.739	0.009	−0.0002 to 0.019	0.055
Clinical model + low peak grip strength^c^	0.427	0.124 to 0.729	0.006	0.012	0.0027 to 0.021	0.011
Clinical model + slow gait speed^a^	Reference			Reference		
Clinical model + PhA	0.344	0.035 to 0.654	0.029	0.026	0.003 to 0.05	0.029
Clinical model + low SPPB^b^	Reference			Reference		
Clinical model + PhA	0.411	0.103 to 0.718	0.009	0.023	−0.0003 to 0.047	0.053
Clinical model + SPPB	Reference			Reference		
Clinical model + PhA	0.428	0.121 to 0.736	0.006	0.024	0.0023 to 0.046	0.031
Clinical model + low peak grip strength^c^	Reference			Reference		
Clinical model + PhA	0.321	0.012 to 0.631	0.042	0.025	0.0003 to 0.049	0.047
Clinical model + slow gait speed^a^ + low peak grip strength^c^	Reference			Reference		
Clinical model + slow gait speed^a^ + low peak grip strength^c^ + PhA	0.365	0.058 to 0.672	0.020	0.022	0.0001 to 0.043	0.049

*Note*: Clinical model: age, male, left ventricular ejection fraction, estimated glomerular filtration rate, N‐terminal pro‐B‐type natriuretic peptide ≥ median. Missing data: 10‐m gait speed, *n* = 2; SPPB, *n* = 13; grip strength, *n* = 7. Abbreviations: CI, confidence interval; IDI, integrated discrimination improvement; NRI, net reclassification improvement; PhA, phase angle; SPPB, short physical performance battery.

^a^ Slow gait speed: preoperative gait speed < 1.0 m/s.

^b^ Low SPPB: preoperative SPPB score ≤ 9.

^c^ Low peak grip strength: male < 28 kgf and female < 18 kgf.

## Discussion

This study demonstrated that PhA was associated with a long‐term prognosis in patients who underwent cardiovascular surgery, even after adjustment by multivariate analysis for potential confounders (cardiac and renal function). Additionally, PhA provided more prognostic information compared with physical performance measures. The risk factors associated with PhA identified in this study were older age, the presence of inflammatory diseases, poor nutritional status and increased NT‐proBNP levels. This study is clinically significant because it shows the usefulness of PhA in predicting long‐term mortality and provides preliminary information on the clinical interpretation of PhA in patients undergoing cardiovascular surgery.

The association between PhA and mortality risk after cardiovascular surgery is consistent with previous reports on other inflammatory diseases,^30^, ^31^ although the clinical interpretation of PhA may vary among diseases. The prognostic impact of the PhA has also been investigated in terms of cardiovascular disease^9^, ^10^; however, evidence regarding the association between the PhA and long‐term prognosis after cardiovascular surgery is still unclear. To this end, we analysed 858 patients and found a relationship between PhA and long‐term post‐discharge prognosis after cardiovascular surgery. This study provides further evidence by demonstrating the potential of the PhA for risk stratification in patients undergoing cardiovascular surgery.

PhA showed significant improvement in predicting the long‐term prognosis after cardiovascular surgery. This improvement was greater than that when other physical function indicators were added to the predictive model, although PhA is strongly correlated with physical performance.^11^, ^32^, ^33^ The additive prognostic values of physical function measurements may be derived from preoperative sarcopenia and physical frailty measures, which have been reported to be associated with long‐term prognosis.^1^ In addition, PhA may have provided further prognostic value by reflecting systemic health status, including malnutrition and inflammation, as well as physical status.^8^ To the best of our knowledge, this is the first report to demonstrate the superior prognostic capability of PhA compared with muscle strength or physical performance tests, which are established predictors of the prognosis of cardiovascular surgery.^1^

There are several possible mechanisms underlying the relationship between the PhA and all‐cause mortality. First, PhA may reflect the quality of skeletal muscle function, such as density or increased intramuscular adipose tissue quality,^34^, ^35^ which has been reported to be associated with prognosis after cardiac surgery^36^ and transcatheter aortic valve replacement.^37^ Furthermore, the association between intramuscular adipose tissues is reportedly related to insulin resistance via oxidative stress and chronic inflammation^38^, ^39^ and is likely to explain the link between PhA and prognosis in patients with cardiovascular diseases. Second, PhA negatively correlated with NT‐proBNP, possibly indicating a more severe cardiovascular condition. NT‐proBNP has been reported to be a key marker of PhA in a previous study,^25^ and an independent relationship between NT‐proBNP and PhA was observed in the present study, supporting this hypothesis. Finally, preoperative systemic health conditions may be associated with the post‐discharge prognosis. In this study, PhA was negatively associated with older age, the presence of cancer and diabetes and poor nutritional status. The results of this study are in line with those of previous studies that reported the determinants of PhA.^32^, ^40^, ^41^, ^42^ However, PhA predicted long‐term mortality even after adjusting for these confounders and showed an increased predictive value compared with that shown by conventional prognostic factors such as physical function indicators, suggesting the specific prognostic value of PhA. In addition, a possible cut‐off value of 4.5° for all‐cause mortality after cardiovascular surgery in this study was similar to a previous study that examined post‐operative short‐term prognosis.^11^ Further studies are required to advance the understanding of the biological and clinical interpretations of PhA and to identify the optimal cut‐off value for risk stratification in clinical practice.

BIA measurement is non‐invasive and can be easily conducted in routine clinical practice at a relatively low cost. In addition, BIA is feasible for patients with preoperative health conditions that limit exercise load, such as symptomatic heart failure and angina pectoris, as it can be performed in the standing or supine position. However, longitudinal changes in PhA have not been well documented in patients with cardiovascular diseases. Previous studies reported in healthy subjects have shown that physical activity^43^ and resistance training^44^ improved PhA. The clinical significance and modifiability of PhA through comprehensive cardiac rehabilitation, including exercise training, educational counselling for disease management and nutritional interventions, need to be examined to consider PhA as a therapeutic target.

This study has several limitations. First, because this was a single‐centre retrospective study, the generalizability of the findings should be carefully discussed. Second, inflammatory parameters, such as C‐reactive protein and interleukin‐6 (IL‐6), were not available for all patients and were, therefore, not included in the analysis. This limits the interpretation of the association between PhA and study outcomes. Third, the study population was composed of a homogeneous group of Japanese patients. Considering the influence of age, sex and race on PhA,^45^ the generalizability of the findings should be confirmed by subgroup analyses or in other racial populations in future studies. Finally, owing to the nature of the observational design, the causal relationship between PhA and study outcomes cannot be addressed. In order to elucidate the causal relationship between PhA and prognosis, it is necessary to examine the impact of changes in PhA due to interventions on prognosis. Nevertheless, the clinical significance of this study lies in showing the value of PhA in predicting long‐term post‐discharge prognosis in patients undergoing cardiovascular surgery using a relatively large cohort, compared with previous studies.

The findings of this study suggest that the PhA is a useful predictor of long‐term mortality in patients undergoing cardiovascular surgery. In addition to the simplicity of measurement, the superior prognostic value of PhA compared with other physical performance tests allows for accurate post‐operative risk stratification with a single measure. PhA can also be measured in inpatients with activity limitations due to symptomatic and/or preoperative conditions, suggesting the potential for making preoperative assessment more widely applicable.

## Conflict of interest statement

Kenichi Shibata, Masataka Kameshima, Takuji Adachi, Hisako Kito, Chikako Tanaka, Taisei Sano, Mizuki Tanaka, Yoriyasu Suzuki, Mototsugu Tamaki and Hideki Kitamura declare that they have no conflict of interest.

## Supporting information


**Figure S1.** Cutoff value from the receiver operating characteristic curves for Phase angle.

**Figure S2.** Kaplan–Meier survival curves for cumulative mortality after cardiovascular surgery divided by Phase angle cutoff of 4.5°.
