# Supplementary figures and images for: Association between preoperative phase angle and all‐cause mortality after cardiovascular surgery: A retrospective cohort study
Source: J Cachexia Sarcopenia Muscle. 2024 Jun 10;15(4):1558–67. doi: 10.1002/jcsm.13514 (PMC11294016; doi:10.1002/jcsm.13514)

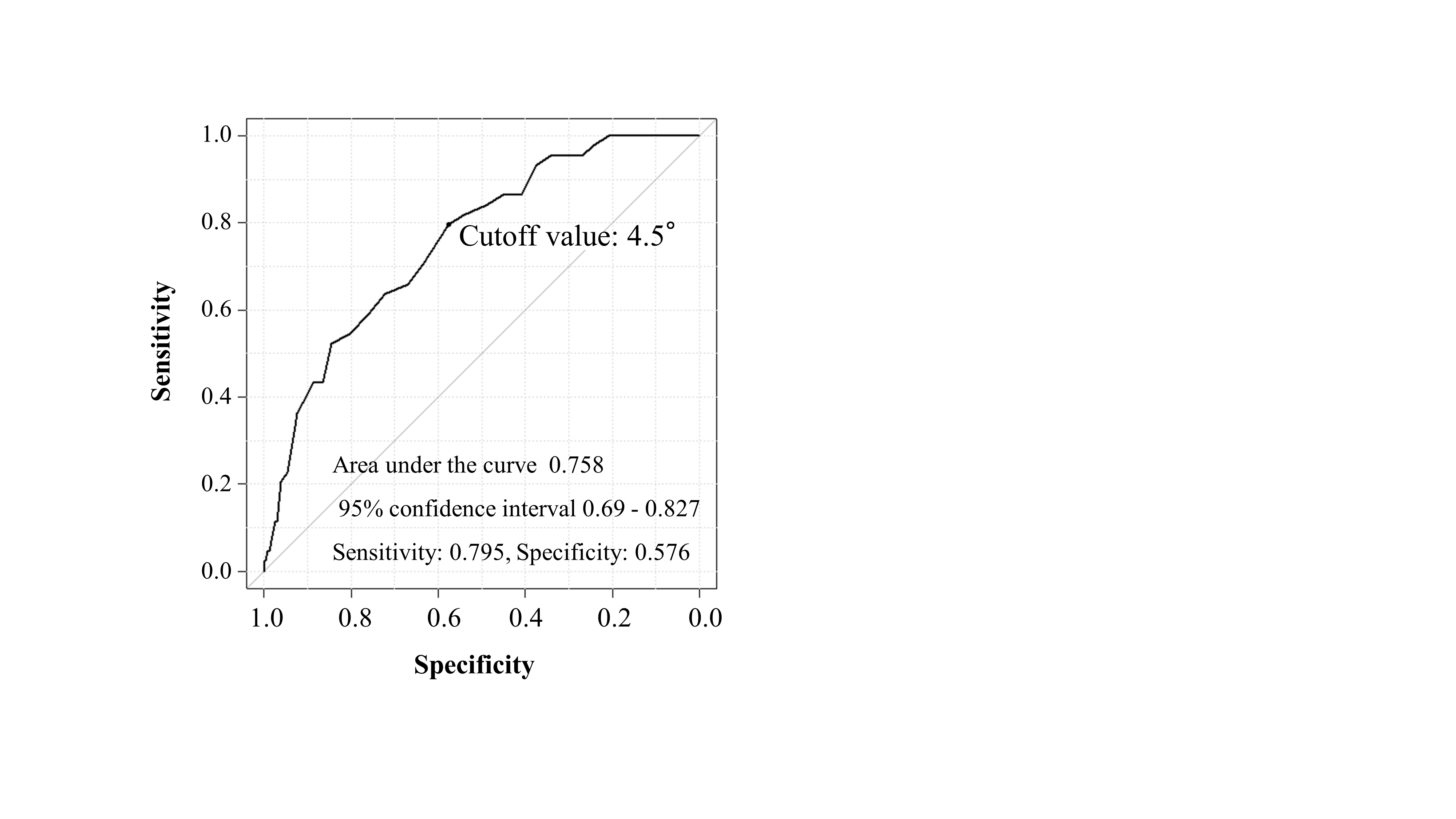

Supplement: Supplementary file 1 — Figure S1. Cutoff value from the receiver operating characteristic curves for Phase angle. [file JCSM-15-1558-s002.tif]

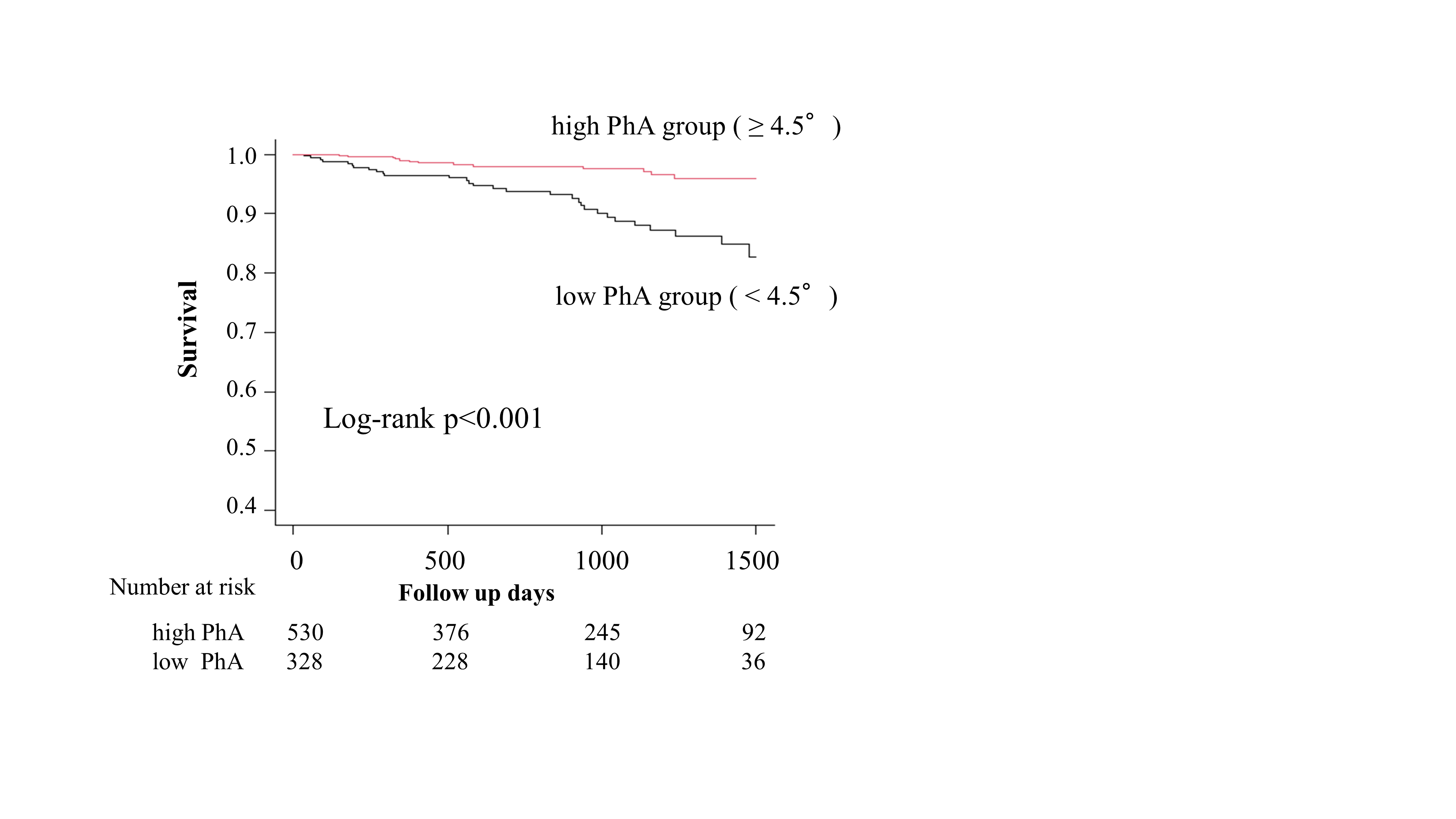

Supplement: Supplementary file 2 — Figure S2. Kaplan–Meier survival curves for cumulative mortality after cardiovascular surgery divided by Phase angle cutoff of 4.5°. [file JCSM-15-1558-s001.tif]
